# Advanced Glycation End Products: A Comprehensive Review of Their Detection and Occurrence in Food

**DOI:** 10.3390/foods12112103

**Published:** 2023-05-23

**Authors:** Lixian Li, Yingjun Zhuang, Xiuzhi Zou, Maolong Chen, Bo Cui, Ye Jiao, Yunhui Cheng

**Affiliations:** 1 School of Food Science and Bioengineering, Changsha University of Science & Technology, Changsha 410114, Chinacyh@csust.edu.cn (Y.C.); 2 School of Food Science and Engineering, Qilu University of Technology, Shandong Academy of Sciences, Jinan 250353, China

**Keywords:** advanced glycation end products, food, detection methods, content

## Abstract

The Maillard reaction (MR) is a complicated chemical process that has been extensively studied. Harmful chemicals known as advanced glycation end products (AGEs), with complex structures and stable chemical characteristics, are created during the final stage of the MR. AGEs can be formed both during the thermal processing of food and in the human body. The number of AGEs formed in food is much higher compared to endogenous AGEs. A direct connection exists between human health and the build-up of AGEs in the body, which can result in diseases. Therefore, it is essential to understand the content of AGEs in the food we consume. The detection methods of AGEs in food are expounded upon in this review, and the advantages, disadvantages, and application fields of these detection methods are discussed in depth. Additionally, the production of AGEs in food, their content in typical foods, and the mechanisms influencing their formation are summarized. Since AGEs are closely related to the food industry and human health, it is hoped that this review will further the detection of AGEs in food so that their content can be evaluated more conveniently and accurately.

## 1. Introduction

Modern food preparation includes hot processing, which can increase food flavor, extend shelf life, and lower the risk of foodborne illnesses [1]. During thermal processing, most commercially treated foods experience the Maillard reaction (MR), a chemical reaction consisting of a complex interaction of free amino groups (derived from amino acids or proteins) and carbonyl groups (derived from sugars) under the influence of heating [2]. A category of hazardous chemically stable substances known as advanced glycation end products (AGEs), formed in the last step of the MR, have complicated structures [3]. Since AGEs are related to a number of diseases, their formation process, types, and methods of quantification should be understood.

The ionic pathway, oxidation pathway, and free radical pathway are the possible means of AGE production in food [1]. These reaction processes can occur inside proteins or in tiny food molecules, with the consequent creation of AGEs of various sizes as the outcome. The complexity of the AGE formation mechanism, their wide range, and the multitude of nutritional components make it difficult to detect these harmful substances in food. Most current studies have measured a few representative AGEs, such as N^ε^-carboxymethyl-lysine (CML) and N^ε^-carboxyethyl-lysine (CEL) [4,5,6,7]. Instruments are typically used to analyze AGEs in food, with the most commonly used method being high-performance liquid chromatography–tandem mass spectrometry (HPLC-MS/MS) [8]. Although the detection accuracy is high, the sample pre-treatment process is very cumbersome and complicated [8].

This review discusses the formation and detection of AGEs in food and their distribution in different foods. A discussion and comparison of detection methods and techniques for analyzing AGEs in food and future research directions are proposed. This review is intended to provide guidance and incentive for further research into AGEs in food and encourage the development of simple, convenient, and accurate methods for their detection.

## 2. Types of AGEs

AGEs are a complex and heterogeneous group of compounds that can be formed from a variety of precursors as a result of the MR [9]. This chapter focuses on the types of AGEs present in foods, including reagents and formation mechanisms, as this is closely linked to AGE detection.

**Figure 1:**
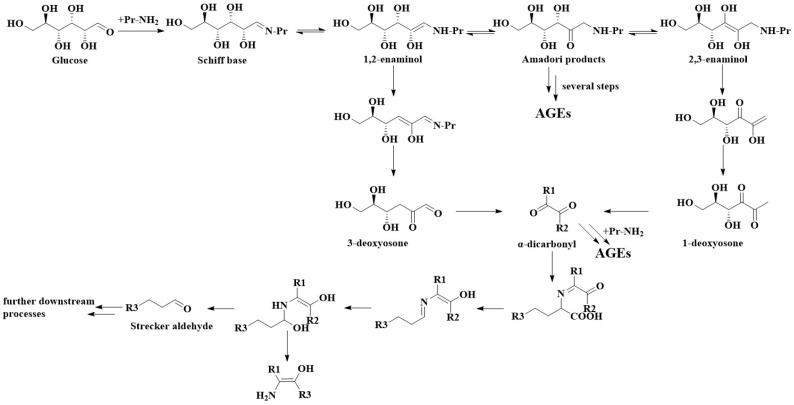
Reaction pathways for AGE formation in foods [10]. Quoted with permission from (Lund, M.N.; Ray, C.A. Control of Maillard Reactions in Foods: Strategies and Chemical Mechanisms. J. Agric. Food Chem. 2017, 65, 4537–4552. https://doi.org/10.1021/acs.jafc.7b00882 (accessed on 12 May 2023)). Copyright (2017) American Chemical Society.

The reaction pathway for the amine group of protein and glucose to generate AGEs was summarized by Lund and Ray, as shown in Figure 1 [10]. Reducing sugars (glucose, aldose) and protein amine groups form Schiff bases, which can be rearranged by 1, 2-amino alcohols to Amadori products (or Haynes products, in the case of ketoses). The Amadori products can then undergo enolization to yield 1, 2-enamelanol or 2, 3-enamelanol and to form deoxyketones (α-dicarbonyl compounds), which rapidly react with other nucleophiles to form aldehydes of the Strecker group. AGEs are obtained following a series of such reactions.

**Figure 2:**
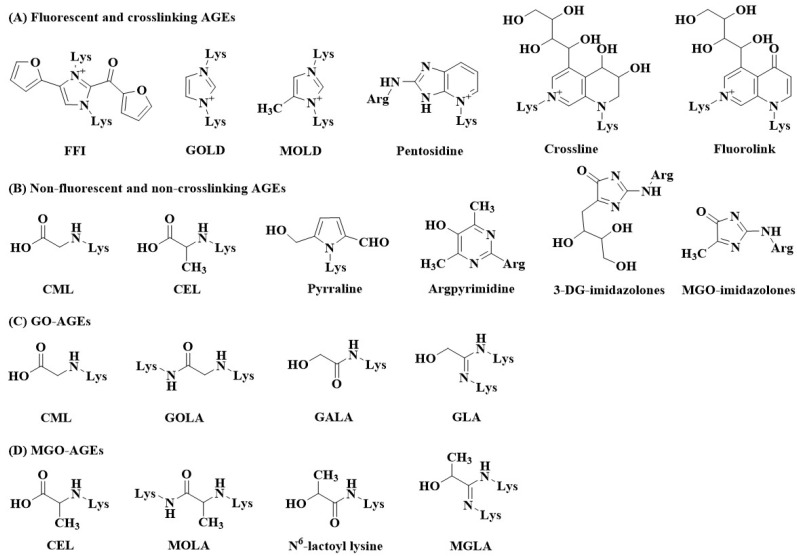
Chemical structural formulas of different types of AGEs.

**Figure 3:**
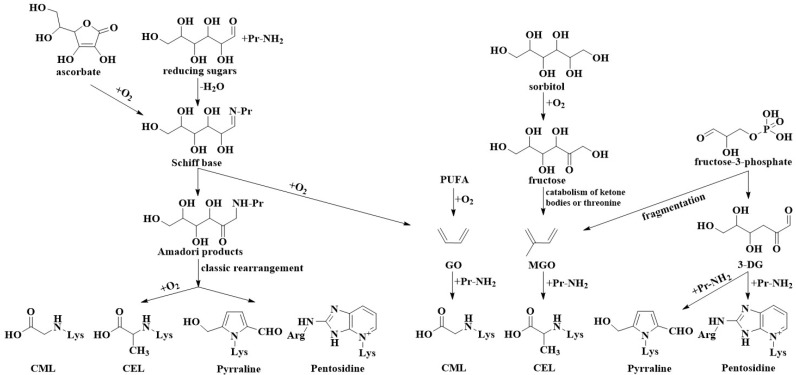
Reaction pathways for common AGE formation in foods.

Regarding the classification of AGEs, there are currently three common classification methods. One is to divide AGEs based on their molecular weight as either low molecular weight (LMW) or high molecular weight (HMW), but the exact boundary between LMW and HMW is not clear [1]. The other classification method divides products into fluorescent and crosslinking AGEs and non-fluorescent and non-crosslinking AGEs [11]. Fluorescent and crosslinking AGEs include pentosidine, crossline, 2-(2-furoyl)-4(5)-(2-furanyl)-1 *H*-imidazole, fluorolink, glyoxal-lysine dimer (GOLD), and methylglyoxal-lysine dimer (MOLD). Non-fluorescent and non-crosslinking AGEs include CML, CEL, pyrraline, argpyrimidine, 3-deoxyglucosone (3-DG)-imidazolones, and methylglyoxal (MGO)-imidazolones. In addition, there are methods to classify AGEs according to the types of dicarbonyl compounds, such as glyoxal (GO)-AGEs and MGO-AGEs [12]. CML, N^6^-glycolyl lysine (GALA), glyoxal-lysine-amide (GOLA), and glyoxal-lysine-amidine (GLA) belong to GO-AGEs, while CEL, methylglyoxal-lysine amide (MOLA), N^6^-lactoyl lysine, and methylglyoxal-lysine amidine (MGLA) belong to MGO-AGEs. The chemical structure of different types of AGEs is shown in Figure 2. Common AGEs are explained in the following sections. The possible formation pathways of CML, CEL, pentosidine, and pyrraline are summarized in Figure 3 [13,14,15,16,17,18,19,20,21,22,23].

### 2.1. CML and CEL

By the middle of the 1980s, scholars believed that all AGE precursor substances were Amadori products and that the outcome of metal-catalyzed oxidative cleavage of the Amadori product to protein was CML [24]. The first AGE, CML, was identified by Ahmed in 1986 [25]. CEL is a CML homologue with a formation pathway that is similar to that of CML [24]. CML/CEL can be formed in several ways, through a reductive sugar and protein reaction, lipid oxidation, and ascorbic acid oxidation. Schiff bases are early MR products (aldose and ketose) formed by the condensation of reducing sugars and amino groups on proteins. Alternatively, they can be formed by cleaving dehydroascorbic acid to form L-threose and then reacting with the lysine residues, followed by a rearrangement of Amadori/Heyns to generate products of Amadori/Heyns, including fructoselysine, lactulosyllysine, and tagatoselysine. Further oxidation of these intermediates leads to CML/CEL formation. Moreover, GO/MGO is a major intermediate in the CML/CEL formation pathway generated by both lipid peroxidation and auto-oxidation of reducing sugars. This reactive dicarbonyl compound reacts further with the lysine residues to form the CML/CEL complex.

### 2.2. Pentosidine

Pentosidine was first discovered as a fluorescent crosslink derived from pentoses formed by arginine and lysine reactions [26]. Next, Grandhee and Monnier [27] explored the mechanism of pentoside, believing that pentosidine can be synthesized in vitro through the reaction of ribose, arginine, and lysine. The precursors of pentosidine include glucose, fructose, and ascorbic acid. [27]. Pentosidine is also thought to be the major fluorophore formed during the non-enzymatic browning of the ribonuclease and lysozyme by glucose [28]. This study showed that pentoside formation occurs most efficiently in the reaction of pentose with lysine and arginine in the model system but is also formed by glucose, fructose, ascorbic acid, Amadori products, and 3-DG as well as other sugars [28]. Notably, pentosidine does not form from peroxidized polyunsaturated fatty acid or malondialdehyde [28].

### 2.3. Pyrraline

The glycosylated lysine is initially obtained by the reaction of glucose with lysine and then degraded to produce the 3-DG. 3-DG reacts with free amino groups again to form pyrraline, which is a rate-limiting step in the pathway of pyrraline formation, and the lower pH promotes the occurrence of this pathway [29].

The formation of pyrraline was studied with a kinetic model of the MR model system of glucose and lysine and postulated to be a combination of 3-DG or MGO and lysine. The results showed that the activation energy of the 3-DG pathway was significantly lower than that of the MG pathway. Kinetic studies have shown that 1, 4-dicarbonyl compounds were cyclized to form pyrrole, and pyrraline was more amenable to generation via the 3-DG pathway [30].

## 3. Measurement of AGEs in Food

There are many kinds of AGEs with complex structures and different contents. This chapter focuses on the detection methods of AGEs, including detection principles, application areas, advantages, and limitations. Current methods for the detecting of AGEs in foods fall mainly into two categories: instrument analysis and immunoassay analysis. Instruments used in these processes include HPLC-DAD (diode array detector) [31], HPLC-FLD (fluorescence detector) [32], HPLC-UV (ultraviolet detector) [33], HPLC-MS/MS [4], UPLC (ultra-high-performance liquid chromatography)-MS/MS [34,35,36], and GC (gas chromatography)-MS [37,38,39]. The most commonly used instrumental analysis method is HPLC-MS/MS, while the primary immunoassay method is ELISA [40,41,42,43,44,45].

### 3.1. Instrument Analysis

#### 3.1.1. HPLC-Detectors

HPLC is usually used in series with a detector to determine AGEs. A list of commonly used HPLC methods, application areas, and detection markers is detailed in Table 1.

HPLC-DAD is used to determine the content of pyrraline [46,47] in food because the DAD detector can detect substances with ultraviolet absorption. HPLC-DAD can accurately detect the content of pyrraline with good linearity, but the sample needs to be pre-treated, generally by enzymatic hydrolysis and acid hydrolysis [46,47]. Pepsin is usually used for enzymatic hydrolysis [47], and hydrochloric acid is mainly used for acid hydrolysis [46,47]. In recent years, HPLC-DAD has been regularly used with a C18 column to determine pyrraline in dairy products [31,46]. Poojary et al. [47] developed a method to detect pyrraline with the ACQUITY HSS T3 Column (100 mm × 2.1 mm, 1.8 μm) configured by UPLC-DAD. This method’s detection limit was found to be as high as 0.50 μg pyrraline/g protein. A UV detector with the advantages of low noise, wide linear range, and good selectivity is also used. While not very sensitive to ambient temperature, changes in mobile phase composition, and flow rate fluctuations, the detection limit is high. Hellwig and Henle [66] used HPLC-UV to determine formyline and pyrraline quantitatively and found that the UV absorption mode differs greatly from the standard. Further research is needed to confirm whether solid phase extraction is suitable for the samples’ purification in this case.

HPLC-FLD is commonly used to detect fluorescent and crosslinking AGEs, such as pentosidine. The method shows high selectivity, high sensitivity, and good reproducibility but only responds to fluorescent AGEs. Non-fluorescent AGEs need to be derived into a fluorescent substance. The use of FLD to detect the content of CML requires hydrolysis and derivatization of the sample. Milk samples should be degreased with n-hexane and reduced with sodium borate before hydrolysis [4,6]. Meat products could be defatted with the degreasing solvents chloroform and methanol before reduction [67]. Hydrolysis is usually carried out with 6 M hydrochloric acid at 110 °C, and derivatization can be achieved by various reagents. The most commonly used reagents are o-phthalaldehyde and 2-mercaptoethanol [68].

HPLC-MS is the most widely used method to measure CML and CEL. However, it is subject to strong interference peaks from the matrix and ion suppression effects, and standard addition and isotope dilution methods are often used to compensate [69]. Ochi [70] used nonafluoropentanoic acid (NFPA) as an ion pair reagent to successfully achieve good retention of CML on a reversed-phase C18 column with good compatibility with HPLC-MS. Sun et al. [71] used water extraction to prepare samples for the analysis of free AGEs. Trichloroacetic acid was used to precipitate proteins to prevent column damage.

CML and CEL content in minced pork was determined by Zhang et al. [51] using the Atlantis silica HILIC column (150 × 2.1 mm, 3 μm) in an HPLC-MS/MS configuration with a low limit of detection of 4 to 5 μg/L for CML and 12 to 15 μg/L for CEL. Subsequent applications were also applied to silver carp surimi sausages [52] and pork tenderloin and offal [7]. When Troise et al. [33] used an HILIC column (75 mm × 2.1 mm, 2.6 µm) to measure CML and CEL in cookies, CML and CEL values > 20 ng/mg could be detected. Xiao et al. [4] used HPLC-MS/MS to configure a Hypercarb guard column (2.1 mm × 10 mm, 5 μm) tandem with a C18 column (4.5 mm × 150 mm, 5 μm) in order to detect the presence of CML in dairy products with a limit of detection of 0.1 μg/kg. Bai et al. [60] developed a method for determining the CML and CEL content in mutton using a Hydro-RP80A column (250 mm × 2 mm, 4 μm). Detection limits were achieved at 3.6 ng/mL for CML and 1.9 ng/mL for CEL. UPLC-MS/MS is also used to measure AGEs in food. The UPLC system can withstand higher pressures and maintain good separation results at lower flow rates [8].

#### 3.1.2. GC

**Figure 4:**
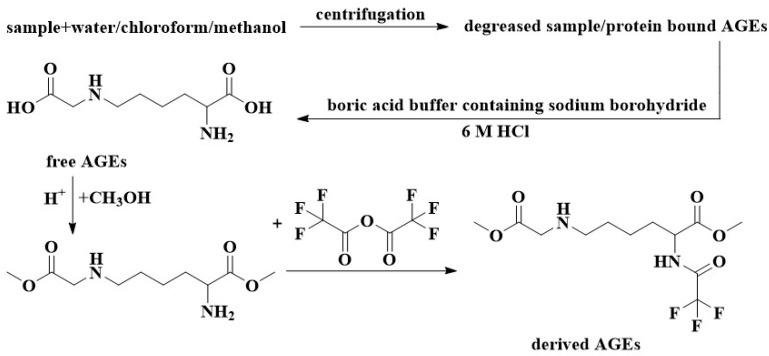
Sample pretreatment for the GC-MS method.

GC is often connected in tandem with MS to detect AGEs. GC requires the pre-treatment of samples, usually degreasing, reduction, hydrolysis, and derivatization, as shown in Figure 4 [37,72]. A mixed solution of water/chloroform/methanol is often used to achieve the purpose of degreasing by centrifugation. The reduction often uses a boric acid buffer containing sodium borohydride, and the sample is often placed in 6 M HCl for acid hydrolysis. Derivatization usually involves esterifying the carboxyl group with methanol under strong acid conditions and then acylating the amine group with trifluoroacetic anhydride [72]. Charissou et al. [73] used GC-MS to determine the CML content in commercial milk powder, biscuits, salmon, and beef. The detection limit was 0.1 ng/mg protein. The quantification limit was 1 ng/mg protein, with no matrix effect. However, the derivatization process before the GC-MS measurement is time-consuming. Malgorzata et al. [74] used GC equipped with a flame ionization detector to measure CML in buckwheat samples, and this method required only acid hydrolysis of the samples.

### 3.2. Immunoassay Analysis

#### ELISA

ELISA can be used as a rapid means of detecting AGEs and can simplify the process of testing samples. ELISA is currently widely used in the medical field to measure AGEs [75], and there are also examples of the application of ELISA in the food field. Fluorescent AGEs such as pentoside and non-fluorescent AGEs such as CML can be detected by ELISA. The principle of ELISA and the advantages and disadvantages of ELISA in the food field will be discussed in the following.

The basic principle of ELISA involves the immobilization of the antigen or antibody and the enzyme labeling of the antigen and antibody [76]. First, the antibody is bound to the surface of a solid phase carrier. The test antigen is added, and then the antigen–antibody-specific immune reaction is carried out with the corresponding enzyme-labeled antibody, generating an antibody-test antigen-enzyme-labeled antibody complex. The enzyme’s substrate reacts to produce a colored product. The quantity of antigen to be tested is directly proportional to the quantity of stained product; therefore, the amount of antigen can be calculated based on absorbance. The principle of antibody measurement is similar to this. With the development of ELISA, commercial ELISA kits have become common. Specific operation of the ELISA kits can be performed as per the manufacturer’s instructions. Commonly used antibodies and their application fields are listed in Table 2.

In 2004, Goldberg et al. [78] used ELISA to detect the CML content in 250 types of foodstuffs, with a well-characterized anti-CML monoclonal antibody (4G9). Results were expressed as units of AGE per mg of protein or lipid. The author stated that the results of the ELISA assay are preliminary and provide a starting point for developing a research database. The systematic food analysis method can obtain more accurate measurements. Charissou et al. [73] compared ELISA and GC-MS in the determination of dairy products. The anti-CML monoclonal antibody 4G9 was used, and the competitive ELISA had a sensitivity of 5 ng/mL CML. When using ELISA rather than GC-MS, the CML concentration in the liquid sample was almost 10 times higher. The difference between the two methods is greatest for hydrolyzed liquid samples. While ELISA lacks specificity compared with GC-MS, it is rapid, low-cost, and has low detection limits [73]. Uribarri et al. [79] used ELISA to determine the content of AGEs in 549 foods using a monoclonal anti-CML antibody (4G9) and an anti-MG monoclonal antibody (3D11 mAb). Results were expressed as AGE kilounits/100 g of food and nmol/100 g or nmol/100 mL of food. Compared to the anti-MG monoclonal antibody, the sensitivity of the method using the monoclonal anti-CML antibody was better [79]. Tareke et al. [80] used an AGE-specific antibody (mouse monoclonal 4G9) conjugated to horseradish peroxidase. The CML level in the three porridge samples analyzed by ELISA was about 56% of the level detected by LC-MS. The relative deviation of ELISA is fairly large [80]. Three different commercially available ELISA kits were used by Gómez-Ojeda et al. [45]. ELISA-1 was a human CML-AGE ELISA kit, while ELISA-2 was an assay kit for competitive inhibition of total AGEs in human serum. The authors believed that ELISA appears to be particularly suitable for the analysis of products with a relatively high concentration of CML/AGEs, but these methods are less precise, and the difference in the concentration of CML in the same food prepared by different cooking treatments cannot be detected at a statistical level by ELISA. These methods are obviously affected by the matrix effect [45]. Prosser et al. [44] used a commercial CML ELISA kit to measure CML in infant formula. The results obtained in this experiment were consistent with the results of many studies using the ELISA method.

It is recommended that nearly all current commercial ELISA kits be used for clinical sample analysis, but few are used for food analysis. Antibodies such as anti-pentosidine, anti-3-DG-imidazolone, anti-CML, anti-CEL mAb, anti-pyrraline, anti-CMA, and anti-human RAGE (receptor for advanced glycosylation end products) mAbs have been widely used in the medical field [75]. In the future, these antibodies can be considered for use in the food field for ELISA.

### 3.3. Other Methods

In addition to the standard methods above, others can be used to determine the content of AGEs. Klostermeyer and Henle [81] used ion exchange chromatography in conjunction with a photodiode array to determine pyrraline. This method allows pyrraline to be quantified at levels < 500 μg/kg protein. Capillary electrophoresis was used by Dalin and Dutta [82] to analyze the in vitro MR of glyceraldehyde, glucose, and fructose and the inhibition of glyceraldehyde self-condensation under physiological conditions using naked capillary and 214 nm UV detection. Birlouez-Aragon et al. [83] used front-face fluorescence to determine the CML content in infant milk powder.

### 3.4. Comparison of the Detection Techniques

There are many methods currently used to detect AGEs. Compared with other instrument analysis methods, HPLC-MS/MS can identify and quantify target analytes with high accuracy and high resolution and does not require sample derivatization [69]. HPLC-MS/MS is suitable for the determination of AGE content in various foods. However, HPLC-MS/MS is a bit more expensive than other methods.

GC-MS is chiefly used for the determination of AGE content in meat, dairy, and cereal products. However, when using HPLC-MS and GC-MS to determine the CML content of porridge, it was found that the standard deviation of GC-MS was ±28, and the relative standard deviation was 10%, while the standard deviation measured by HPLC-MS was ± 6 and the relative standard deviation was 5%. Therefore, the precision and stability of GC-MS are lower than those of HPLC-MS [80]. In addition, the application of GC-MS is less extensive than that of HPLC-MS.

Compared to instrumental analysis, ELISA appears to be particularly suitable for studying products with relatively high concentrations of CML/AGEs. ELISA has been used to measure the AGE content of various foods, which facilitates the establishment of a preliminary database. This method, however, is less accurate than LC-MS and is significantly impacted by matrix effects. When using the ELISA method, the number of AGEs in fatty foods may be overestimated and the level of AGEs in starchy foods may be underestimated [45,84]. Nevertheless, ELISA is a good choice for the determination of AGE content in a large number of samples in a short time. Furthermore, it should be emphasized that commercial kits currently used in the study are recommended for clinical sample analysis, while there are actually no commercial kits for food analysis.

## 4. Levels of AGEs in Common Foods

According to the formation mechanism of AGEs summarized above, AGEs mainly exist in foods rich in proteins, sugars, and fats. Many articles focus on AGEs in foods, and reports of AGE content in hundreds of foods exist. This section focuses on the recent research progress on AGEs in common foods.

### 4.1. Cereal Products

Cereal products are a common food type consumed in daily life. The MR is an important chemical reaction in bakery products, and the safety of cereal products obtained through baking has received much attention. Cereal products reported to be linked to AGEs in recent years mainly include breads and cookies. There have also been studies on tortillas, sweetheart pastries, and instant noodles. At present, research on AGEs in cereal products primarily focuses on CML, CEL, and methylglyoxal-derived hydroimidazolone-1 (MG-H1), and LC-MS/MS is generally used for measurement (Table 3). Raw materials, ingredients, processing time, and processing temperature are the main factors that affect the content of AGEs in cereal products.

#### 4.1.1. Raw Materials

The AGE content in cereal products is affected by the raw materials, in connection with the content of protein, amino acids, lipids, and dietary fiber. Zilic et al. [85] reported the CML and CEL contents of sweet cookies made with whole grain flour of eight wheat genotypes (bread wheat, durum wheat, soft wheat, durum wheat, triticale, rye, hulless barley, and oats) and four maize genotypes (white, yellow, and red standard seed corn, and blue popcorn). CML and CEL contents were found to be positively correlated with the total protein and total amino acid contents of the flour. Hence, the CML and CEL contents of the cookies produced from whole wheat oat flour were the highest because of their high total lysine and protein content [85]. Since okara is known to have various health benefits, it was added to cookies to replace 15% wheat flour; however, the degree of browning and the CML content in these cookies significantly increased with a stronger MR [86]. This increase was related to the protein and lipid of the okara, as unsaturated fats were thermally oxidized during roasting, promoting the formation of CML. Moreover, the authors claimed that this phenomenon was primarily related to the presence of approximately 50% of the insoluble dietary fiber in okara, which reduced the aqueous activity of the biscuits and promoted MR during cooking [86]. Gómez-Ojeda et al. [45] found that the content of CML in flour tortilla and corn tortilla was significantly lower than that of whole wheat bread, possibly because the impact of corn and wheat processing during the tortilla processing on the CML formation was lower than that of the production of other foods based on these grains. Cheng et al. [87] measured CML and CEL content in nine cereal products (chicken cookies, baby biscuits, white lotus paste mooncakes, almond biscuits, cookies, bread, sweetheart pastry, fried breadsticks, and instant noodles). The content of CML and CEL in the bread and biscuits category was significantly higher than that in sweetheart pastry and instant noodles. This may be a result of the high processing temperature and low moisture content of the bread and biscuits, which facilitated the MR and formation of AGEs [87]. In addition, CML and CEL were the highest in baby biscuits. Thus, attention should be paid to controlling the content of AGEs during the processing of baby food.

#### 4.1.2. Ingredients

In order to enrich the taste and improve the flavor of cereal products, various kinds of ingredients are used frequently. The effects of fructose, glucose, honey, and bananas on the number of AGEs in cookies have been explored by Treibmann et al. [88]. Under the same conditions, there was no significant difference in the formation of CML when glucose and fructose were added, although the highest content of MG-H1 and CEL was observed in the biscuits with the highest fructose content, possibly because fructose was more likely to produce MGO [88]. In addition, the effects of the addition of sucrose, butter, and egg fluid on the formation of AGEs in butter cookies were investigated [89]. Sucrose supplementation did not significantly impact free CML and CEL content, which may be a result of the low free lysine content in the biscuits. On the other hand, when the amount of added sucrose was greater than 10 g (25% of the flour weight), both protein-bound CML and protein-bound CEL showed a significant increase with the increase in sucrose addition. The increase in sucrose content promoted the production of α-dicarbonyl compounds (GO, MGO, and 3-DG), leading to the production of more protein-bound AGEs. As for butter, when its addition was greater than 15 g (37.5% of the flour mass), there was a significant increase in the content of protein-bound AGEs. A greater amount of butter contributed to the formation of α-dicarbonyl compounds, which was associated with the formation of short-chain α-dicarbonyl compounds through lipid oxidation reactions, leading to an increase in AGEs bound to proteins. As the addition of whole egg fluid increased, the content of free CML and CEL initially increased and then decreased, suggesting that free CML and CEL were involved in the subsequent reactions as the protein-bound CML and CEL gradually increased. The egg fluid provided sufficient protein and free amino acids for butter biscuit baking. When the amount of amino supplementation was large during cooking, the MR was dominant and may have taken precedence over the caramelization reaction.

#### 4.1.3. Processing Time and Temperature

Processing time and temperature have been shown to have significant influences on the AGE content of cereal products. The effect of baking times (7, 10, and 13 min, at 180 °C) on the AGE content of sweet cookies was investigated [85]. CML and CEL content increased with increasing heating duration, which was linked to a significant increase in the content of the dicarbonyl compounds after baking. The influences of baking time (8–15 min) and temperature (130–180 °C) on the formation of AGEs in butter cookies were investigated by Hu et al. [89]. As cooking temperature and cooking time increased, CML and CEL levels initially increased and then decreased, and changes in protein-bound AGEs lagged behind those in free AGEs. Most α-dicarbonyl compounds (GO, MGO, and 3-DG) positively correlated with baking temperature and baking time. As intermediates, α-dicarbonyl compounds led to the formation of AGEs; however, the pattern of GO and MGO formation was inconsistent with AGEs, indicating that the MR is continuously releasing active dicarbonyl compounds, and that AGEs could be involved in the downstream reaction. Jin et al. [90] studied the changes in the CML content of cookies over a broader range of baking times (1.5–31 min) and temperatures (155–230 °C). CML was found to be more likely to form at a range of relatively elevated temperatures (205–230 °C), and an environment of higher temperature and lower humidity strongly promoted CML formation. CML content was highest in cookies baked at 230 °C for 1.5 min. After an extended cooking period in a bakery, the CML content decreased owing to degradation or further reactions. Deep baking, extended baking times coupled with high baking temperatures, seems to be a potential option for significantly reducing the CML content in cookies but would result in unacceptable organoleptic properties. Çelik et al. [62] reported the content of CML and CEL in bread crust heated for a different amount of times (5, 15, and 30 min) at 200 °C. The concentration of CML in the crust did not change significantly, while the CEL content showed an overall downward trend after heating for more than 5 min. On the other hand, the content of the HMW fractions of AGEs (MW > 10,000 Da) was significantly higher than that of CML and CEL in the bread crust type samples, which was related to the HMW fraction of the melanoidin-containing samples formed at the end of the MR.

### 4.2. Meat Products

At present, AGE content is reported in fish, pork, beef, and chicken, most of which include CML, CEL, MOLD, MOLA, and MGLA as listed in Table 4. Currently, the content of AGEs in meat products is chiefly determined by GC-MS, ELISA, and HPLC-MS/MS. Studies have shown that the content of AGEs in meat products is mainly related to the heat treatment process, the type of meat and food additives.

#### 4.2.1. Heat Treatment Process

Common cooking methods for meat include frying, roasting, and stir-frying, accompanied by high-temperature conditions [94,95]. Eggen and Glomb [12] reported that AGEs in raw pork were mainly GO-derived CML. Grilling led to increases in MGO-derived AGEs such as CEL, N^6^-lactose lysine, MOLD, and MOLA, while GO-derived AGEs such as CML, GOLD and GOLA had fewer increases. This suggested that the grilling process resulted in a significant rise in MGO quantity. Even though the content of GO in raw meat was approximately 4-fold higher than that of MGO, after grilling, the MGO content was 2-fold higher than GO content. On the contrary, some scholars have found that the level of CML in processed pork was 2–3 times than that of CEL [96], which may be a result of sample type and shape as well as overall processing conditions including temperature and time [12]. Studies have shown that when the internal temperature of roast beef reached 90 °C and 100 °C, the content of fluorescent compounds increased significantly. Nevertheless, the formation of CML was not detected until the internal temperature reached 300 °C [91]. When the effects of temperature and time on the formation of CML and CEL during the thermal processing of sausage were investigated [92], their levels did not change at 70 °C and 90 °C, but rose rapidly at 110 °C, and increased about two times at 130 °C. The accumulation of α-dicarbonyl compounds was thought to accelerate CML and CEL formation. The rapid increase in α-dicarbonyl content at elevated temperatures might result from the combined effect of the MR and lipid oxidation. At the same time, a high-temperature caused the peptide bond to break, expanding the protein structure and exposing active amino acids, which favored CML and CEL production [92]. Even at the same temperature, different heat treatments would also lead to varying levels of AGEs in meat products. More CML was produced by grilling and frying than baking [67]. Furthermore, the CML content in the outer layer of the fried meat was significantly greater than that in the middle layer, which may be related to more water-soluble precursors transferred to the meat surface during the firing process. In addition, the frequency of turning during frying also affected CML formation in meat products. The CML content of the single-turn meat was significantly higher than that of the multiple-turn meat when the internal temperature of the samples was the same. This was possibly due to the accelerated loss of water-soluble precursors by multiple turning.

#### 4.2.2. Type of Meat

CML was reported to form in beef, pork, chicken, and fish during grilling [67]. Although no significant differences between beef, pork, and chicken were found, grilled salmon and tilapia contained less CML than the other muscle samples. Salmon and tilapia had higher moisture content and pH and lower lipid and protein content than beef, pork, and chicken, which may contribute to some extent to differences in CML levels reported. Niu et al. [7] investigated the changes in CML and CEL levels in 10 commercial brands of pork tenderloin, heart, liver, and kidney before and after the heat treatment. The mean values of free CML and CEL in the macroscopic kidneys were higher than those in the other organs, which could be explained by the fact that the kidneys are the primary excretory organs of vertebrates, making them important sites for the clearance and metabolism of AGEs. For example, in the case of free AGEs, AGEs bound to small peptides or amino acids could be rapidly filtered and cleared by the kidneys, leading to significantly higher levels of free AGEs in the kidney compared with other organs. In contrast, the content of heat-induced AGEs bound to proteins in the liver and kidney was lower than that in the spine and heart, which may be attributed to the greater accumulation of antioxidants in the kidneys and in the liver inhibiting or slowing the formation of heat-induced AGEs. At the same time, more pro-oxidants, such as metal ions, may accumulate in the heart and promote heat-induced AGE formation.

#### 4.2.3. Ingredients and Additives

The effect of salt on the formation of AGEs in meat is complicated. Some investigators have proposed that adding salt could make muscle fibers expand with water, improving the water-locking ability of meat, making it less prone to water loss during heating, thereby inhibiting the generation of both CML and CEL [92]. Others have suggested that salt may accelerate lipid oxidation in pork and beef, promote iron ion release, and have pro-oxidative activity [97]. Meanwhile, high concentrations of salt made the lysine residue ε-NH_2_ on the protein surface more vulnerable to attack, resulting in an increase in protein carbonyl content [98]. Li et al. [63] found that the CML and CEL content in roast beef patties increased with the addition of salt and polyphosphate (PP) caused by the rising surface temperature of the patties. The addition of polyphosphate could improve the hardness of the roast beef patties by forming a hard shell on the surface of the patty during baking, obstructing the evaporation of surface water and making the surface temperature rise rapidly.

Lu et al. [92] explored the effects of nitrite on AGEs in sausages and found that nitrite supplementation had a strong inhibitory effect on CML production in the samples. One possible reason for this is that nitrite can inhibit protein oxidation and lipid oxidation in meat, thus reducing AGE production. In addition, nitrite potentially forms a large amount of NO and NO_2_ in sausages, which is more readily involved in oxidation reactions than lipid and protein. Furthermore, nitrite was able to form stable complexes with iron ions, reduce heme iron release, and then inhibit free iron oxidative catalysis [97].

The effect of acetic acid on the production of CML and CEL in commercially sterilized pork was investigated [93]. In pork heated to 121 °C for 10 min, the addition of 0.5% acetic acid was able to promote CML formation but slowed the formation of CML and CEL in pork heated for 30 min relative to the sample in the absence of acetic acid. The authors interpreted this as the phenomenon by which low pH could also enhance the Amadori rearrangement and the degradation of Amadori rearrangement products catalyzed by acid. On the other hand, the decrease in CML and CEL content may be due to decreased nucleophilicity of the amino groups under acidic conditions, thereby reducing the rate of reaction of the initial step in the MR between the amino group of the amino acid or protein and the carbonyl group of the reducing sugar.

### 4.3. Dairy Products

The dairy products reported in recent years have mostly included milk powder and liquid milk. Among them, AGEs chiefly include CML, CEL, pyrraline, MG-Hs, glyoxal-hydroimidazolone (GO-Hs), GOLD, MOLD, GOLA, and GALA, which were determined by various methods as shown in Table 5. Studies have shown that the content of AGEs in dairy products is mainly related to the type, sterilization process, and storage conditions of dairy products.

#### 4.3.1. Type of Dairy Products

Different dairy products contain different amounts of AGEs. Xiao et al. [4] determined the content of free and protein-bound CML in seven types of dairy products (28 liquid milk, 26 milk powder, 6 condensed milk, 4 milk fat, 9 cheese, 6 ice cream, and 3 whey proteins). Free CML in liquid milk was lower than protein-bound CML, and correlation analysis showed that protein in liquid milk did not correlate with CML. The concentration of CML in unsweetened condensed milk was higher than that of sweetened condensed milk, which may be related to the processing of condensed milk. Free CML and protein-bound CML concentrations were highest in whey protein samples, possibly owing to the abundance of protein in powdered protein drinks, which made CML form more readily. There was no significant difference in the CML content between natural cheese and processed cheese (several natural cheeses, melted and processed). In addition, CML was found in ice cream (low-temperature production), and the authors believed that the CML might have come from the raw material of the ice cream.

#### 4.3.2. Sterilization Methods

There are different opinions regarding the effect of sterilization on the content of AGEs in liquid milk. Xiao et al. [4] measured the CML content of 28 liquid milk products (9 pasteurized milk, 18 sterilized milk) commercially available in China. The average contents of free and protein-bound CML in the five pasteurized milks were higher than those of all the sterilized milk, which may be related to the longer time of pasteurization treatment than that of sterilization. Some studies determined that high-temperature treatment formed more AGEs. Li et al. [100] quantitated the CML and CEL pasteurization-treated milk (low-temperature long-time (LTLT), high-temperature short-time (HTST)) and sterilization-milk (ultra-high-temperature (UHT), in-bottle sterilization (BS)) that are applicable in China. CML and CEL levels in UHT and BS milk were significantly greater than those in LTLT and HTST milk samples. It was believed that temperature played a more important role in the formation of CML and CEL than the duration of heat treatment.

The increase in CML, CEL, and pyrraline in high-temperature-treated whole milk powder was greater than that in low-temperature-treated samples. It was supposed that the high-temperature preheating of raw milk further promoted the MR, resulting in more AGEs being formed. In addition, the changes in CML and CEL in whole milk powder and milk reconstituted under different preheating conditions during milk powder processing (low-temperature pasteurization (LT), high-temperature pasteurization, ESL, UHT, and in-container sterilization (CS)) were studied by Li et al. [101]. The CML and CEL contents of the ESL, UHT, and CS samples were higher than that of the milk powder samples at low preheating levels. This result can be explained by the fact that preheating at low temperatures (<100 °C) causes a dynamic equilibrium between furfural and AGEs, leading both to accumulate slowly. High-temperature (>100 °C) preheating treatments would promote the loss of 5-methyl-furfural with active-reaction properties, leading to the generation of either aldehyde and ketone compounds or AGEs with significantly increased CML and CEL contents. Furthermore, the greatest increase in CML and CEL content was observed in CS preheated milk powder and reconstituted milk. It was found that as the temperatures of heat treatment increased, the rate of AGE formation increased.

#### 4.3.3. Storage Conditions

Dairy storage conditions also influence AGEs content, and adverse storage conditions further promote the occurrence of the MR. The reactivity between the sugar and the amine moiety increases with the temperature increases. As the reactivity between the sugar and the amine moiety increases, the MR reaction becomes more intense, leading to an increase in CML concentration in infant formula [40]. The change in the CEL content was similar to the trends mentioned previously. MR products in milk stored at 20 °C, 30 °C, and 40 °C for 1 year were determined by Zhang et al. [103], including CML, CEL, MG-H1/3, G-H1/3, and pyrraline. The concentrations of CML and CEL increased with increasing storage temperatures (40 °C). At the end of storage, however, CML and CEL concentrations in milk stored at 30 °C and 40 °C decreased compared to pre-storage levels, which may be because of the further conversion of CML and CEL to other MR products. Pyrraline was more easily formed at 40 °C, and its concentration continuously increased during storage at all storage temperatures, which correlated positively with the loss of the α-dicarbonyl components. At the 40 °C storage condition, MG-H1/H3 had a content that was 2–5 times greater than that of G-H1/H3, indicating that MGO could react more readily with Arg residues than GO [103]. Humidity (52%) was also found to facilitate the formation of CML in whole milk powder over a 200-day analysis period [40]. The storage time had a significant effect on CML and CEL content as well [6]. For whole milk powder, the CML content slowly increased by 59–94% in the first 12 months of storage, and the rate of formation accelerated from the twelfth month. Compared to month 12, the CML content at month 18 increased by 0.9–1.5 times, possibly owing to more α-dicarbonyl compounds formed by the accelerated lipid oxidation in later storage [6,40].

### 4.4. Coffee, Cocoa and Tea Products

Cocoa, coffee, and tea are three major non-alcoholic beverages enjoyed worldwide in countries with different cultural backgrounds. In these beverages, the AGEs recently studied are CML and CEL, determined by LC-MS/MS.

#### 4.4.1. Coffee Products

Coffee is usually processed by roasting. Liu et al. [107] investigated the effects of roasting temperature (230, 235, 240, and 245 °C) and time (10–18 min) on the CML content of green coffee beans. The CML content of coffee during roasting slowly rose within the first 10 min, then dropped sharply in the next 2 min, then increased again. Baking at 235 °C for 12 min resulted in the lowest CML content. Loaec et al. [108] measured CML in 24 commercial coffee substitutes and 12 instant coffees. CML content varied between 0.17 and 47 mg/kg and increased in proportion to the protein content of the sample used in this study. The CML content in the instant coffee samples was significantly higher than that in the commercial coffee substitute samples because of the relatively high protein content in the instant coffee.

#### 4.4.2. Cocoa Products

Taş and Gokmen [109] conducted a study on the effects of alkali treatment and baking conditions on the CML content of cocoa. The concentration of α-dicarbonyl compounds in the alkali-treated cocoa was higher than in the water-immersed cocoa and untreated cocoa. During the subsequent roasting process, the CML concentration of the alkali-treated cocoa greatly increased to 125 μg/kg of defatted cocoa, almost 2-fold higher than that of the water-immersed and untreated cocoa samples. Under the same pre-treatment, after baking for 30 and 60 min at 135 °C and 150 °C, the concentration of CML in the cocoa samples hardly changed.

#### 4.4.3. Tea Products

Jiao et al. [110] determined the CML and CEL content in 99 tea samples (44 green tea, 7 oolong tea, 41 black tea, and 7 dark tea) from different origins. Black tea and dark tea had higher CML and CEL content, whereas green tea and oolong tea had lower CML and CEL content. The results suggested that the processing of tea significantly influences the CML and CEL content. During the withering process of black tea, the hydrolytic activity of proteases, amylase, and invertases increased with the loss of water, and then amino acids and simple sugars increased through enzymatic reactions. As a result, the levels of active precursors for CML and CEL increased, leading to more production of CML and CEL. For dark tea, the thermal effect of pile fermentation and heat treatment resulted in a high content of CML and CEL. On the contrary, green tea had less CML and CEL because of the low degree of processing involved. Although the CML and CEL content of oolong was relatively low, the degree of withering and fermentation of oolong tea was positively correlated with them. Additionally, the catechin content in green and oolong tea was higher than that of black and dark tea, and catechins were verified to trap GO and MGO.

## 5. Conclusions and Perspectives

In the past few years, there has been substantial progress in research involving AGE detection methods. Instrumental analysis methods have matured, and commercial ELISA kits are more widely used. However, some challenges remain. First, there are various AGE determination methods, but the results obtained by different methods are difficult to compare. Second, the kinds of AGEs measured by ELISA kits are limited, most of which are used to measure CML, though this method is convenient and fast. Finally, changes in processing methods can appropriately reduce the levels of AGEs, but their effects are limited, considering the flavor of food.

Research on AGEs is crucial for human health, so a rapid and accurate detection method of AGEs in food is necessary. The production of AGEs during food processing, transportation, and storage can be better controlled based on the timely data of AGEs. In the meantime, a corresponding database of AGEs in food can be established according to the dietary needs of different groups, and corresponding dietary recommendations can be given to improve human health.

## Figures and Tables

**Table 1:** AGEs detected by different instruments.

Method	Column	Application Field	Marker	Limit of Detection	References
HPLC-DAD	Reverse-phase (RP)-C18 column (4.6 mm × 250 mm, 5 μm)	cookies	fluorescent AGEs		[31]
HPLC-PDA	Sunfire C18 column (4.6 mm × 250 mm, 5 µm)	baked milk and baked yogurt	pyrraline		[46]
UPLC-DAD	ACQUITY HSS T3 column (100 mm × 2.1 mm, 1.8 μm)	milk	pyrraline	0.50 μg/g protein	[47]
HPLC-FLD	C18 reverse-phase column (250 × 4.6 mm, 5 μm)	human urine and plasma	pentosidine	1 nM	[32]
HPLC-MS	Hydro-RP 80 Å LC column (150 × 4.6 mm)	dicarbonyl-BSA model system	CEL, CML, and MG-H1		[48]
HPLC-MS/MS	Hypercarb guard column (2.1 mm × 10 mm, 5 μm) tandem a C18 column (4.5 mm × 150 mm, 5 μm)	dairy products	CML	0.1 μg/kg	[4]
HPLC-MS/MS	Atlantis silica HILIC column (150 mm × 2.1 mm, 3 μm)	pork tenderloin and offal	CML and CEL		[7]
HPLC-MS/MS	Intrada amino acid column (2.0 mm × 150 mm, 3 μm)	model systems with whey protein	CML, G-H3, and MG-H3		[49]
HPLC-MS/MS	Phenomenex BioZen Peptide XB-C18 column (2.1 × 150 mm, 1.7 μm)	black tea	MG-Hs, GO-Hs, CML, CEL, and argpyrimidine		[50]
HPLC-MS/MS	Atlantis silica HILIC column (150 × 2.1 mm, 3 μm)	minced pork	CML and CEL	CML: 4–5 µg/L; CEL: 12–15 µg/L	[51]
HPLC-MS/MS	Atlantis silica HILIC column (150 mm × 2.1 mm, 3 μm)	silver carp surimi sausages	CML and CEL		[52]
HPLC-MS/MS	Sequant-ZIC-HILIC column (250 mm × 4.6 mm, 5 μm)	roasted nuts and seeds	CML and CEL		[53]
HPLC-MS/MS	Phenomenex Kinetex C-18 column (50 × 2.1 mm, 1.7 µm)	human saliva	CML, CEL, MG-H1, and pyrraline		[54]
HPLC-MS/MS	Phenomenex Synergi Hydro-RP column (150 mm × 2 mm, 4 μm)	MR model systems containing ascorbic acid	pyrraline, CML, and CEL		[55]
HPLC-MS/MS	RP-18 (Xselect HSS T3, 250 mm × 3.0 mm, 5 μm)	bread	CEL, N^6^-lactoyl lysine, MG -H1, MG-H3, CEA, CML, GALA, CMA, G-H3, N^6^-formyl lysine, N^6^-acetyl lysine, pyrraline	CEL: 0.05 mg/kg; N^6^-lactoyl lysine: 0.02 mg/kg; MG-H1: 0.07 mg/kg; MG-H3: 0.05 mg/kg; CEA: 0.06 mg/kg; CML: 0.06 mg/kg; GALA: 0.06 mg/kg; CMA: 0.17 mg/kg; G-H3: 0.11 mg/kg; N^6^-formyl lysine: 0.03 mg/kg; N^6^-acetyl lysine: 0.01 mg/kg; pyrraline: 0.12 mg/kg	[56]
LC-MS/MS	Hypercarb column (100 × 2.1 mm, 5 μm)	human milk	CML and CEL		[57]
LC-MS/MS	BEH Amide column (2.1 × 100 mm, 1.7 μm)	fried sturgeon fillets	CML and CEL		[58]
LC-MS/MS	Hydro-RP 80A LC column (2 × 150 mm, 4 μm)	cheeses	CML and CEL		[59]
LC-MS/MS	Hydro-RP80A column (250 mm × 2 mm, 4 μm)	mutton	CML and CEL	CML: 3.6 ng/mL; CEL: 1.9 ng/mL	[60]
LC-MS/MS	Phenomenex Synergi 4 μ Hydro-RP80A column (250 mm × 2 mm, 4 μm)	beef	CML and CEL		[61]
UPLC-MS/MS	X-Bridge C18 (4.6 mm × 150 mm, 5 μm)	baked products	CML and CEL		[34]
UPLC-MS/MS	BEH Amide column (100 × 2.1 mm, 1.7 μm)	fish cakes	CML, CEL, and MG-H1		[35]
UPLC-MS/MS	SB-C18 column (2.1 × 50 mm, 1.8 μm)	soy sauce, corn juice	CML		[36]
UPLC-MS/MS	T3 column (150 mm × 4.6 mm × 3 µm)	bread	CML and CEL	CML: 0.75 μg/kg; CEL: 2.5 μg/kg	[62]
UPLC-MS/MS	T3 column (150 mm× 2.1 mm, 3.5 μm)	roasted beef patties	CML and CEL	CML: 0.052 μg/g; CEL: 0.098 μg/g	[63]
UPLC-MS/MS	BEH Amide column (100 × 2.1 mm, 1.7 μm)	sterilized milk	CML	CML: 0.05 mg/kg	[64]
UPLC-MS/MS	HILIC column	antler velvet	CML and CEL	CML: 4.1 ng/g; CEL: 4.3 ng/g	[65]

CML: *N^ε^*-(carboxymethyl)lysine. CEL: *N^ε^*-(carboxyethyl)lysine. GLAP: glyceraldehyde-derived pyridinium compound. MG-Hs: methylglyoxal-hydroimidazolones. GO-Hs: glyoxal-hydroimidazolones. CEA: N^7^-carboxyethyl arginine. CMA: N^7^-carboxymethyl arginine. GALA: N^6^-glycoloyl lysine.

**Table 2:** Types of antibodies used in ELISA.

Antibodies	Application Field	Limit of Detection	References
4G9	dairy products	CML: 5 ng/mL	[73]
6D12	AGEs-protein		[77]
4G9	food		[78]
4G9 and 3D11	food		[79]
4G9	porridge samples		[80]

**Table 3:** AGE levels in different cereal products.

Influencing Factors	Detection Methods	Cereal Products	AGE Content	References
Raw materials	LC-MS	flour tortilla, corn tortilla	CML: 2.09–2.51 μg/g	[45]
	HPLC-FLD	bread (wheat, brown bread, rye bread, pumpernickel, and crispbreads)	CEL: 2.1−8.1 mg/kg; N^6^-lactoyl lysine: 0.15−0.72 mg/kg; CEA: 7−19 mg/kg; MG-H1: 13−27 mg/kg; MG-H3: 0.28−0.72 mg/kg; CML: 4.5−10.4 mg/kg; GALA: 0.23−0.59 mg/kg; CMA: 1.1−1.6 mg/kg; G-H3: 3.8−5.5 mg/kg; N^6^-formyl lysine: 2.8−5.6 mg/kg; N^6^-acetyl lysine: 1.3−3.6 mg/kg; pyrraline: 28−78 mg/kg	[56]
	UPLC-MS	bread crust (whole wheat, refined wheat, whole einkorn, whole corn, whole rye, whole oat)	CML: 15–140 mg/kg; CEL: 30–200 mg/kg	[62]
	LC-MS/MS	cookies (bread wheat, durum wheat, soft wheat, hard wheat, triticale, rye, hulless barley and hulless oat, white-, yellow- and red-colored standard seeded corn and blue-colored popping corn)	CML: 6.3–47.4 mg/kg; CEL: 2.3–38.9 mg/kg	[85]
	HPLC-MS/MS	cookies (okara cookies, cellulose cookies, pea fiber cookies, chitosan cookies)	CML: 6.32–22.84 mg/kg	[86]
	UPLC-QqQ-MS/MS	chicken cookies, baby biscuit, white lotus paste mooncakes, almond biscuit, cookies, bread, sweetheart pastry, fried breadstick, instant noodles	CML: 4.48–35.88 mg/kg; CEL: 1.99–14.49 mg/kg	[87]
Ingredients	UPLC-MS/MS	sponge cakes	protein-bound CML: 80 mg/kg; free CML: 160 mg/kg; protein-bound-CEL: 15 mg/kg; free CEL: 40 mg/kg	[34]
	HPLC-MS/MS	cookies (sucrose, glucose, fructose, honey, banana, invert sugar syrup)	CML: 1.71–42 mg/kg; CEL: 1.541–53 mg/kg; MG-H1: 8.8–218 mg/kg	[88]
	LC-MS/MS	cookies (sucrose, butter, egg liquid)	free CML: 50–250 μg/kg; free CEL: 80–220 μg/kg; protein-bound-CML: 4–6 mg/kg; protein-bound-CEL: 20–70 mg/kg	[89]
Processing time	UPLC-MS	bread crust (5–30 min)	CML: 15–140 mg/kg; CEL: 30–200 mg/kg	[62]
	LC-MS/MS	cookies (7–13 min)	CML: 6.3–47.4 mg/kg; CEL: 2.3–38.9 mg/kg	[85]
	LC-MS/MS	cookies (8–16 min)	free CML: 0–60 μg/kg; free CEL: 100–400 μg/kg free; protein-bound-CML: 0–20 μg/kg; protein-bound-CEL: 100–450 μg/kg	[89]
	UPLC-MS	cookies (0–10 min)	CML: 0–350 mg/kg	[90]
Processing temperature	LC-MS/MS	cookies (130–180 °C)	free CML: 0–100 μg/kg; free CEL: 50–700 μg/kg; protein-bound-CML: 0–100 μg/kg; protein-bound-CEL: 100–700 μg/kg	[89]
	UPLC-MS	cookies (155–230 °C)	CML: 0–350 mg/kg	[90]

**Table 4:** AGE levels in different meat products.

Influencing Factors	Detection Method	Meat Products	AGE Content	References
Heat treatment process	HPLC-MS/MS	raw pork	CML: 3.0 mg/kg; CEL: 0.9 mg/kg; GALA: 0.9 mg/kg	[12]
		roast pork	CML: 9.2 mg/kg; CEL: 40 mg/kg; GALA: 5 mg/kg; MOLA: 6 mg/kg; GOLA: 1.2 mg/kg; MOLD: 0.5 mg/kg; GOLD: 0.25 mg/kg; MGLA: 0.2 mg/kg	
	HPLC-FLD	meat (fried, boiled and baked)	CML: 1.07–21.84 µg/g	[67]
	GC-MS	fried fish nuggets (180 °C, 4–6 min)	CML: 29.26–59.17 mg/kg	[72]
	UPLC-MS	beef (60–300 °C); (grilled, fried, boiled and baked)	CML: 0.8–13.4 mg/kg	[91]
	UPLC-MS/MS	sausage (70–130 °C; 1–4 h)	CML: 2–6.58μg/g; CEL: 6–16.32 μg/g	[92]
Type of meat	HPLC-MS/MS	pork tenderloin, hearts, livers and kidneys	CML: 0.41–2.56 mg/kg; CEL: 0.22–2.56 mg/kg; protein-bound CML: 2.53–7.06 mg/kg; protein-bound CEL: 1.60–4.83 mg/kg	[7]
	HPLC-FLD	beef steak, pork top loin, chicken breast, salmon, tilapia	CML: 1.07–2.05 µg/g	[67]
Food additives	UPLC-MS/MS	sausage (0–5 g/100g salt; 0–15 mg/100g NaNO_2_)	CML: 2.37–6.52 µg/g; CEL: 5.54–6.56 µg/g	[92]
	HPLC-MS/MS	commercially sterilized pork (acetic acid, ethanol, and NaCl)	CML: 2.29–15.35 mg/kg; CEL: 2.75–39.33 mg/kg	[93]

**Table 5:** AGE levels in different dairy products.

Influencing Factors	Detection Method	Dairy Products	AGE Content	References
Sterilization process	LC-MS	pasteurized milk and sterilized milk	free CML: 8.23–14.02 μg/kg; protein-bound CML: 2.58–5.56 mg/kg	[4]
	LC-MS/MS	whole milk powder (low-temperature long-time (LTLT), high-temperature short-time pasteurization (HTST)) and high-heat process (ultra-pasteurization (ESL), ultra-high-temperature (UHT) treatments, and in-bottle sterilization (BS))	CML: 20–40 mg/kg; CEL: 5–15 mg/kg; pyrraline: 0.15–0.20 mg/kg	[6]
	LC-MS/MS	milk (low/high-temperature)	CML: 2–40 nmol/mg; CEL: 0.2–1.1 nmol/mg; pentosidine: 16–21 pmol/mg	[99]
	UPLC-MS/MS	milk (LTLT, HTST, UHT and BS)	CML: 2–4 mg/kg milk; CEL: 0.4–0.8 mg/kg milk	[100]
	UPLC-MS/MS	milk powder and reconstituted milk (LT, HT, ESL, UHT, CS)	CML: 3–10 mg/kg; CEL: 0.75–2.75 mg/kg	[101]
	UPLC-MS/MS	milk powder	free CML: 0.36–5.22 mg/kg; free CEL: 0.12–1.80 mg/kg; free pyrraline: 0–0.37 mg/kg; protein-bound CML: 21.07–128.34 mg/kg; protein-bound CEL: 13.78–56.62 mg/kg; protein-bound pyrraline: 2.85–81.48 mg/kg	[102]
Storage conditions	LC-MS/MS	whole milk powder (0–18 months)	CML: 20–150 mg/kg; CEL: 5–55 mg/kg; pyrraline: 0.15–0.55 mg/kg	[6]
	ELISA	infant formulas (25 °C, 37 ℃; 0–28 days)	CML: 500–600 ng/mL	[40]
	UPLC-MS/MS; UPLC-DAD	UHT milk (22 ℃; 12 months)	CML: 25–50 μM; CEL: 2.5–13 μM; G-H1/3: 22–35 μM; G-H2: 4–8 μM; MG-H1/3: 15–35 μM; MG-H2: 0–0.6 μM	[47]
	ELISA	infant formula (7–28 d; 32% RH, 57% RH, 75% RH; 25 °C, 37 °C)	CML: 550–600 ng/mL	[103]
	LC-MS	UHT milk (6 months; 20 °C)	CML: 1.794 ng/mL; CEL: 7.29 ng/mL; G-H1/3: 1.63 ng/mL; G-H2: 2.49 ng/mL; MG-H1/3: 3.82 ng/mL; GOLD: 32.8 ng/mL	[104]
	UPLC-MS; HPLC-UV	infant formulas (65 °C; 48 days)	CML: 280 mg/kg; CEL: 180 mg/kg; MG-H: 190 mg/kg; GO-H: 280 mg/kg; GOLD: 40 mg/kg; MOLD: 20 mg/kg; pyrraline: 60 mg/kg	[105]
	UHPLC-MS/MS	whey proteins (35 °C; 7, 14, 21 days)	CML: 25–150 μM; CEL: 1–2 μM; MG-H1: 4–15 μM	[106]
Types of dairy products	LC-MS	liquid milk, milk powder, condensed milk, milk fats, cheese, ice cream, and whey protein	free CML: 0.11–32.71 μg/kg; protein-bound CML: 0.01–134.28 mg/kg	[4]
	UPLC-MS/MS; HPLC-PDA	baked milk and baked yogurt	CML: 0.2–1.7 mg/kg; CEL: 0.06–0.1 mg/kg	[46]

## Data Availability

Not applicable.
